# A retrospective analysis of health systems in Denmark and Kaiser Permanente

**DOI:** 10.1186/1472-6963-8-252

**Published:** 2008-12-11

**Authors:** Anne Frølich, Michaela L Schiøtz, Martin Strandberg-Larsen, John Hsu, Allan Krasnik, Finn Diderichsen, Jim Bellows, Jes Søgaard, Karen White

**Affiliations:** 1 Copenhagen Hospital Corporation, Bispebjerg Bakke 23, Bispebjerg Hospital, 2400 Copenhagen NV, Denmark; 2 Institute of Public Health, University of Copenhagen, Øster Farimagsgade 5, Building 15, 1014 Copenhagen K, Denmark; 3 Center for Health Policy Studies, Kaiser Permanente, 2000 Broadway, Oakland, CA 94612, USA; 4 Institute of Public Health, University of Copenhagen Øster Farimagsgade 5, Building 9, 1014 Copenhagen K, Denmark; 5 Care Management Institute, Kaiser Permanente, One Kaiser Plaza, 16th Floor, Oakland, CA 94612, USA; 6 Danish Institute for Health Services Research Dampfærgevej 27–29, 2100 Copenhagen Ø, Denmark; 7 Institute for Global Health, University of California/San Francisco, 50 Beale Street, San Francisco, CA 94105, USA

## Abstract

**Background:**

To inform Danish health care reform efforts, we compared health care system inputs and performance and assessed the usefulness of these comparisons for informing policy.

**Methods:**

Retrospective analysis of secondary data in the Danish Health Care System (DHS) with 5.3 million citizens and the Kaiser Permanente integrated delivery system (KP) with 6.1 million members in California. We used secondary data to compare population characteristics, professional staff, delivery structure, utilisation and quality measures, and direct costs. We adjusted the cost data to increase comparability.

**Results:**

A higher percentage of KP patients had chronic conditions than did patients in the DHS: 6.3% vs. 2.8% (diabetes) and 19% vs. 8.5% (hypertension), respectively. KP had fewer total physicians and staff compared to DHS, with134 physicians/100,000 individuals versus 311 physicians/100,000 individuals. KP physicians are salaried employees; in contrast, DHS primary care physicians own and run their practices, remunerated by a mixture of capitation and fee-for-service payments, while most specialists are employed at largely public hospitals. Hospitalisation rates and lengths of stay (LOS) were lower in KP, with mean acute admission LOS of 3.9 days versus 6.0 days in the DHS, and, for stroke admissions, 4.2 days versus 23 days. Screening rates also differed: 93% of KP members with diabetes received retinal screening; only 46% of patients in the DHS with diabetes did. Per capita operating expenditures were PPP$1,951 (KP) and PPP $1,845 (DHS).

**Conclusion:**

Compared to the DHS, KP had a population with more documented disease and higher operating costs, while employing fewer physicians and resources like hospital beds. Observed quality measures also appear higher in KP. However, simple comparisons between health care systems may have limited value without detailed information on mechanisms underlying differences or identifying translatable care improvement strategies. We suggest items for more in-depth analyses that could improve the interpretability of findings and help identify lessons that can be transferred.

## Background

While promoting effective, affordable health care is a universal goal, health care systems vary considerably in their approach. Comparisons of health systems could help identify successful strategies and models for achieving this goal and provide useful benchmarks for change [1].

A structural reform of the Danish healthcare system (DHS) was undertaken in 2007 with the goal of performance improvement and increasing effectiveness of care. We looked to other health systems for transferable practices, and Kaiser Permanente (KP) had been described as providing effective care at costs comparable to those of the UK National Health Service [2]. Of particular interest was KP's experience with developing care for chronic conditions, for which prevalence rates are high and increasing in Denmark.

Many existing studies and reports generate a 'landscape' view of health systems, presenting data in various categories from four to eight or more nations [3-8]. For health policymakers, these broad comparisons highlight trends and identify high performers, but leave many questions unanswered about the interpretability and utility of the information. For instance, determinating how much of the observed variation reflects delivery and financing approaches, rather than unmeasured variations in the underlying population and medical practices, can be challenging and often missing. When modifiable health system structures do explain outcome differences, the transferability into other contexts and specific implementation strategies often are unknown [9,10].

Only a few comparisons attempt to examine two systems along several dimensions [11]. In this paper, we compare two systems along six dimensions–population, professional staff, delivery system, utilisation patterns, quality measures, and medical costs–using typically available secondary data. By using data available in most health care system settings, our comparison attempts to develop a widely applicable health policy tool. We use a national health care system and a sub-national system as examples, examining the extent to which our comparison helps us understand their differences and similarities. We also suggest a framework for health systems comparisons that supports more robust knowledge.

We compared data from the Kaiser Permanente integrated care delivery system in California (KP), and the Danish Health Care System (DHS). The populations are approximately the same size at 6.1 million and 5.3 million, respectively; however, the enrolled KP population and the geographically-bounded Danish population differ. The KP population generally does not include unemployed individuals and under represents individuals who are elderly, low-income, or handicapped, as they are covered under Medicare or Medicaid. The two systems have comparable benefits and entitlements [12], with a few exceptions. Kaiser Permanente members are only partially covered for inpatient and emergency room admittance, mental health inpatient services, and substance abuse inpatient services.

## Methods

To compare the two health care systems, we focused on aspects of the health care system derived from both the Chronic Care Model and Donabedian's model of structure, process, and outcome [13,14]. We further refined our categories according to the availability of data.

We focused on identifying secondary data sources that were as comparable as possible. KP data came from automated data systems, the national Healthcare Effectiveness Data Information Set [15], published reports [16], and an internal member survey [17]. DHS data came from government ministry reports [18-24], national registries and professional organisations [25,26], published reports [27-29], and Organisation for Economic Co-operation and Development and World Health Organization reports [30-32].

Additionally, to increase comparability, we adjusted the cost data in several ways. First, we converted Danish gross expenditures in Danish krone (DKK) to USD using 2000 purchasing power parities. We then subtracted capital depreciation and profit from gross expenditures to obtain operating expenditures for each system. Dental benefits vary between the systems, so we excluded these costs. We also excluded long-term nursing care expenses from DHS costs, because, while the figures reported to the Organisation for Economic Co-operation and Development include these costs, the care is provided and funded by the municipal social service system. Long-term nursing care for KP was not included, since individuals, supplemental long-term care insurance, or governmental agencies pay for it.

We adjusted the Danish per capita expenditures for differences between the populations in age, education, and income. Danish income data was converted to US dollars using purchasing power parity (PPP) conversion rates. We then stratified Danish health care costs into age, education, and household income categories. By applying the characteristics of the KP population to these stratified costs, we adjusted the per capita Danish costs for differences between the populations.

## Results

### Population

The KP population was younger, better educated, and wealthier on average, compared to the DHS population. A lower percentage of KP members were 65+ years (10.2%) than in the DHS (15.1%) (Table 1). Nearly 95% of KP members had a high school diploma, while less than two thirds did in the DHS. In US dollars, 6.1% of KP members reported annual household incomes below $15,000, compared with 16% in the DHS. Conversely, 18% of KP members reported household incomes higher than US $100,000 per year, compared to only 5% of the Danish population.

**Table 1:** Population characteristics

	Kaiser Permanente (%)	Danish Population (%)
Age in years		
0–4	6.0	6.4
5–15	15.0	13.0
16–44	43.1	40.2
45–64	25.7	25.6
65–74	6.3	8.1
75–84	3.2	5.2
≥ 85	0.7	1.8
		
Educational level		
		
Less than high school	5.3	37.4
		
High school or higher	54.9	42.3
		
Bachelors degree or higher	39.8	20.3
		
Household income in USD (thousands)		
< 15	6.1	16.0
15–25	9.2	14.6
25–35	11.1	13.8
35–50	17.5	15.6
50–65	12.9	17.9
65–80	13.3	11.1
80–100	12.1	6.1
> 100	17.9	4.9

Data on educational level of KP membership is from 2002.

Danish utilisation index is from 2001; index adjusted for age, sex and income where all inhabitants older than 15 years = 100.

Data on household income levels of Kaiser Permanente membership is from 1998.

More KP members reported having chronic conditions than did Danish citizens: 6.3% reported having diabetes mellitus in KP vs. 2.8% in DHS; 19% reported having hypertension in KP vs. 8.5% in DHS; and 1.0% reported having a stroke in KP vs. 0.2% in DHS.

The rates for individual risky behaviours such as excess weight and smoking also varied between the populations (Table 2). Fewer KP members reported smoking on a daily basis than did Danish citizens. While the percentages who were overweight, defined as having a BMI from 25–30, were similar in the two populations, a higher percentage of KP members met the definition of obesity; i.e., BMI > 30.

**Table 2:** Smoking and obesity rates

	Kaiser Permanente 2002 Age ≥ 20 years		DHS population 2000 Age ≥ 16 years		DHS population 2005 Age ≥ 16 years	
Risk factors	Men	Women	Men	Women	Men	Women
Smoking rate (%)	14	11	39	35	32	28
Overweight (%) (BMI between 25 to 30)	43.4	26.0	40	26	41	26
Obese (%) (BMI > 30)	21.9	23.3	10	9	12	11

Data on risk factor prevalence is from the Northern California region only.

### Professional Staff

KP had fewer physicians and total health professionals than did the DHS: 134 physicians and 1,125 health professionals per 100,000 members versus 311 physicians and 2,025 health professionals per 100,000 citizens. Physicians include all types of physicians: residents, physicians, specialists, and general practitioners. Health professionals cover all health professionals except physicians.

### Delivery system

Both systems rely on contractual relationships between individual physicians and the health care delivery system. However, the delivery systems for primary care are quite different. All KP physicians are salaried members of multi-specialty physician groups. In the DHS, specialists are primarily salaried hospital employees, but all primary care physicians (PCPs) are self-employed and receive a combination of capitation and fee-for-service compensation. In addition, 38% of DHS PCPs have solo practices.

### Utilisation patterns

Hospital beds in KP were occupied 270 days per 1,000 persons per year, compared to 814 days per 1,000 persons per year in the DHS. Acute care admission rates showed a similar spread: seven per 1,000 persons per year in KP and 18 per 1,000 persons per year in Denmark.

The length of stay for acute admissions averaged 3.9 days at KP and 6.0 days in Danish hospitals (Table 3). Stroke patients displayed the most remarkable difference in average length of stay. They remained hospitalised an average of 4.26 days at KP, compared to 23 days in Denmark.

**Table 3:** Mean hospital lengths of stay by diagnosis for patients age 65 and over

Diagnoses	KP Days (mean)	DHS Days (mean)
Stroke	4.3	23.0
COPD	3.8	5.1
Coronary bypass	9.8	N/A
AMI	4.4	7.2
Angina pectoris	2.2	4.5
Hip replacement	4.5	9.5
Hip fracture	4.9	12.1
Kidney or urinary bladder infection	3.8	5.0

At KP, cardiovascular angioplasty rates were 25% higher and the rate of coronary bypass grafts was twice that of the DHS. KP also had higher kidney transplantation rates (4.8 per 100,000 population compared to 2.9 per 100,000).

### Quality Processes

KP had higher rates for breast cancer screening (78% vs. 10%), retinal screening among patients with diabetes (93% vs. 46% in the only reporting Danish county), and beta-blocker use among patients with acute myocardial infarction (93% vs. 69%). Screening rates for cervical cancer were roughly comparable at 80% and 75%.

### Medical Costs

Operating expenditures for KP and the DHS were similar at PPP $12,975 million and $12,535 million (Table 4). Per capita expenditures were higher for KP at PPP $1,951, compared to PPP $1,845 for the DHS. Adjusting for different distributions of age, education and income yielded Danish per capita expenditures of PPP $1,480, 24% less costly than at KP.

**Table 4:** Health care expenditures

Category	Kaiser Permanente (2000) US Dollars	Danish Healthcare System (2000) US Dollars
Gross expenditures/revenue adjusted for:	$14 200 m	$12 791 m
Less capital depreciation	- $557 m	- $256 m
Less profit	- $668 m	- 0
Operating expenditures:	$12 975 m	$12 535 m
		
Operating expenditures corrected for different expenditures:	$12 975 m	$12 535 m
Dental care	- $10 m	-$473 m
Special circumstances	- $ 1 065 m	-$278 m
Long-term nursing care		- $ 2 283 m
		
Net expenditures after corrections:	$11 900 m	$ 9 779 m
		
Standardised per capita expenditures (6.1 million people for Kaiser; 5.3 million people for DHS)	$1 951	$1 845
Adjustments for age differences	$1 951	$1 639
Final adjusted per capita expenditure	$1 951	$1 480

‘Special circumstances’ refers to sales, marketing, and malpractice insurance (Kaiser Permanente) and research and development and state-covered malpractice insurance (Danish Healthcare System). For the DHS net expenditures ($9 799m) equals operating expenditures less (dental costs plus long term care, less supplementary private health insurance).

## Discussion

Our comparison revealed intriguing differences between health systems in all the dimensions we explored. However, we were interested not only in the differences themselves, but also in how they could inform policy decisions.

The interpretability of health outcome and efficiency differences was limited by substantial variations between the populations. These variations were both in principle, i.e. an enrolled and a geographic population in two different countries, and empirical. The structures and system outputs also appear to differ between the systems, but not always with consistent patterns. Observed cross-sectional differences between the systems might reflect differences in the timing or pace of similar trends, rather than structural differences. For instance, cross-sectional observation fails to reveal that the trend in the DHS follows the movement within KP from inpatient to outpatient delivery settings. The average length of stay in the DHS decreased from 6.0 in 1993 to 3.8 in 2000 and, over the same time period, the number of hospital beds in the DHS per1000 members of the population decreased from 5.0 to 4.3.

### Populations, behaviours, and disease detection

The two populations have different reported prevalence rates of selected chronic diseases and risk factors. The higher reported disease prevalence in KP could be attributable to several factors, including more aggressive case finding, high rates of obesity and inactivity throughout the US, and, perhaps, differing emphasis on disease prevention between the two systems. While we cannot be certain of the relative contribution of these factors, we believe that the higher prevalence rates in KP cannot be attributed solely to case finding and that the higher real disease prevalence in the KP population is driven largely by social and cultural differences between the US and Denmark.

Unfortunately, variable rates of case finding make it impossible for us to quantify the real differences in disease prevalence or to apply case-mix adjustment to our utilization and cost statistics. Case-mix adjustment would lower KP's costs relative to DHS and further lower its utilization. Drawing conclusions about the differences between populations requires more data on disease patterns and risk factors and also requires data that is more comprehensive, detailed, standardised, and objective (e.g., administrative or derived from electronic health records or population studies that include health examinations). One great challenge to a more detailed comparison is that there is no diagnosis recording in primary healthcare in Denmark.

### Staffing and structure

One of our most striking findings is the more than two-fold difference in the numbers of physicians in the two systems. Several interpretations are possible. KP physicians tend to work more hours, from 40–70 hours per week compared to about 40 hours in the DHS [32,33]. But Denmark may also be lagging behind in transfer of clinical and other tasks from physicians to other health professionals.

The relationship between primary and specialty care is also unclear from these figures and warrants a closer look. Are generalists expected and able to provide some specialty care, for newly diagnosed diabetes, for instance? Or all patients referred to another setting for care? At what point does that referral take place?

The differences in hospital utilisation are also striking. Higher hospital utilisation can reflect failures of primary care [33], greater supply such as more available beds [34], or favourable reimbursement approaches, including use-based payments. Indeed, the total number of hospital beds in each system is important information that supports understanding absolute and excess capacity. While the hospital reimbursement practices are comparable in the two systems, existing utilisation data fail to capture variations in structure and cultural patterns of care. In general, acute care lengths of stay in US hospitals have dropped over the last decade [35]. Additionally, in Denmark, stroke patients receive rehabilitation services in the hospital, while in KP and the United States in general, more of these patients receive some of their care outside of the hospital. The number of beds in alternative care settings and patterns of hospital discharge practices would provide much-needed context for the differing lengths of stay in the two systems.

Higher specialty procedure rates at KP may reflect discrepancies in chronic disease prevalence. If more members do indeed have heart disease, then the higher procedure rates follow. However, supply can also contribute to higher procedure rates, as do prevailing practice patterns [36,37]. Additional data on availability of specialists and specialty care facilities, practice norms, and reimbursement differences are needed. To develop a complete picture of the relative utilisation of levels of care, we would also need indicators of use and access, like primary and specialty care visit rates and wait times for primary and specialty care.

### Modifiable and transferable practices

Readily available data only allowed us to paint a very cursory picture of the two systems. Transferring successful practices requires much more extensive information on elements such as leadership and governance structures, economic and non-economic incentives, the prevailing models of care (including the degree of continuity between settings and over time), and the presence of factors that enable evidence-based practices, such as electronic health records and other technologies.

In addition, the nature of professional responsibilities may vary across health care systems. More data is needed on factors such as the educational preparation, professional roles, and scope of practice of physicians, nurses, rehabilitative therapists, health behaviourists, and other health care professionals.

### Quality documentation, measurement, and outcomes

Our study revealed differences in the quality of care, as measured by process indicators such as conformity to best practice recommendations. An obvious difficulty with our findings is inconsistent reporting throughout the DHS, but quality is a particularly challenging dimension along which to compare health care systems.

International collaborations have attempted to identify quality indicators [38-40]. The Organisation for Economic Co-operation and Development launched the Health Care Quality Indicators project to build on this work and to broaden involvement to all member nations. It identified recommended indicators in five priority areas: diabetes, cardiac care, mental health, patient safety, and primary care and prevention. However, substantial measurement gaps remain in these areas, as do problems with data availability and comparability [41]. Additionally, many current approaches use process-based measures, just as we do, that appear to be weakly, if at all, associated to actual patient outcomes [42]. Adverse event rates, however, do reflect patient outcomes and so could constitute a 'bare minimum' quality indicator as international quality consensus work develops.

### Medical costs, factor costs, and resource use

We found age-, education- and income-adjusted purchasing power parity (PPP) expenditures per person to be 24% higher in KP than in Denmark. We adjusted for differences in age and income distributions and educational levels in KP and DHS, as these factors strongly affect both health status and health care utilization in both countries [43].

Using GDP-PPPs to convert Danish kroner to US dollars, we adjusted for general price level differentials in the two countries [44]. This is the most common currency conversion method, even for sector specific comparisons, such as health care expenditures [45-47].

However, this method does not adjust for differences in health care factor costs that are not parallel to general cost and price levels of the economy. Health care factor costs in the US, and hence in KP, are undoubtedly higher than in Denmark [48,49]. Comparable and reliable physician wage and/or labour income data are hard to find, but we estimate that physician starting salaries in KP are 80% higher than in Denmark and about 40% higher for specialists, except for primary care physicians who are well remunerated in Denmark.

Apart from uncertain data, our wage/remuneration cost comparisons are limited by differential work loads, as noted above, and the two systems being at different stages of transferring tasks from physicians to other professionals.

A broader perspective on our findings of a 24% difference in health care expenditures is that average US health care expenditures per capita (PPP$ 4,570) in 2000 were 92% higher than in Denmark (PPP$ 2,379) [50]. Therefore, it is unlikely that the per capita difference reflects efficiencies in care. However, it is possible that some of the per capita cost difference is due to the higher prevalence of chronic conditions in KP and an emphasis on effective care delivery to patients with chronic illnesses [51].

## Conclusion

The challenges of data availability, comparability, and adjustment methodologies limit the usefulness of simple health systems comparisons for identifying successful strategies for transfer. Table 5, see additional file 1, lists several of the types of information that could improve the interpretability of differences between systems and help identify transferable lessons across systems. For more specific comparisons, additional information and more detailed data are needed.

While examination of the substantial variations in health care delivery across systems and countries holds much promise, deeper knowledge of populations, professional staff, delivery systems, utilisation patterns, quality processes, and costs lays the foundation for identifying differences in health systems that confound findings about the relationships between inputs, structures, quality, and resource use. Without the ability to identify and control for confounding factors, transferring practices across settings is unlikely to occur with any regularity–or with success.

## Competing interests

The authors declare that they have no competing interests.

## Authors' contributions

AF designed the concept and conducts of the study, collected analysed and interpreted data, drafted the manuscript, and participated in the decision to submit it. MLS collected, analysed, and interpreted data and helped to draft the manuscript. MSL collected, analysed, and interpreted data and helped to draft the manuscript. JH: analysed and interpreted data, contributed substantially to drafting the manuscript and completed critical revisions for important intellectual concepts, and participated in the decision to submit it. AK assisted in study design, data analysis and interpretation, critical revision of the manuscript for important intellectual concepts, and decision-making about manuscript submission. FD analysed and interpreted data. JB analysed and interpreted data critical revision of the manuscript for important intellectual concepts. JS analysed and interpreted the economic issues and provided a critical revision of the manuscript for important intellectual concepts. KW collected, analysed, and interpreted data.

## Pre-publication history

The pre-publication history for this paper can be accessed here:



## Supplementary Material

Additional file 1 **Elements of health systems useful for making meaningful comparisons.** Recommended sample types of information in six domain areas for comparing health systems. Click here for file

## References

[B1] Ginsburg JA, Doherty RB, Ralston JF, Senkeeto N, Cooke M, Cutler C, Fleming DA, Freeman BP, Gluckman RA, Liebow M, McLean RM, Musana KA, Nichols PM, Purtle MW, Reynolds PP, Weaver KM, Dale DC, Levine JS, Stubbs JW (2008). Achieving a high-performance health care system with universal access: what the United States can learn from other countries. Ann Intern Med.

[B2] Feachem RG, Sekhri NK, White KL (2002). Getting more for their dollar: a comparison of the NHS with California's Kaiser Permanente. BMJ.

[B3] Blendon RJ, Schoen C, DesRoches C, Osborn R, Zapert K (2003). Common concerns amid diverse systems: health care experiences in five countries. Health Affairs.

[B4] Blendon RJ, Schoen C, Donelan K, Osborn R, DesRoches CM, Scoles K, Davis K, Binns K, Zapert K (2001). Physicians' views on quality of care: a five-country comparison. Health Affairs.

[B5] Hussey PS, Anderson GF, Osborn R, Feek C, McLaughlin V, Millar J, Epstein A (2004). How does the quality of care compare in five countries?. Health Affairs.

[B6] Schoen C, Blendon RJ, DesRoches CM, Osborn R (2002). Comparison of health care system views and experiences in five nations, 2001: findings from The Commonwealth Fund 2001 International Health Policy Survey. Issue Brief (Commonw Fund).

[B7] Brown LD (2003). Comparing health systems in four countries: lessons for the United States. Am J Public Health.

[B8] Organisation for Economic Development and Co-operation (2007). OECD Health Data 2007: Statistics and Indicators for 30 Countries. Paris.

[B9] Rundall TG (2007). Evidence-based management. Hosp Health Netw.

[B10] Shortell SM, Rundall TG, Hsu J (2007). Improving patient care by linking evidence-based medicine and evidence-based management. JAMA.

[B11] Tawfik-Shukor AR, Klazinga NS, Arah OA (2007). Comparing health system performance assessment and management approaches in the Netherlands and Ontario, Canada. BMC Health Serv Res.

[B12] Bilde L, Ankjaer-Jensen A, Danneskiold-Samsoe B (2005). The "Health Benefit Basket" in Denmark: a description of entitlements, actors, and decision-making processes in the curative health sector. Eur J Health Econ.

[B13] Bodenheimer T, Wagner EH, Grumbach K (2002). Improving primary care for patients with chronic illness. JAMA.

[B14] Donabedian A (2003). An introduction to quality assurance in health care.

[B15] National Committee for Quality Assurance (2001). Making an informed choice with HEDIS 2000 performance measures. Kaiser Permanente program overview. Washington DC.

[B16] Ham C, York N, Sutch S, Shaw R (2003). Hospital bed utilisation in the NHS, Kaiser Permanente, and the US Medicare programme: analysis of routine data. BMJ.

[B17] Gordon NP (2004). Kaiser Permanente Characteristics of Members Aged 20 and Over in the Northern California Region Kaiser Permanente Membership as Estimated from the 2002 Adult Member Health Survey. Oakland.

[B18] Ministry of Interior and Health (2006). The health sector in numbers. Health Statistics Copenhagen.

[B19] (2007). Health statistics.

[B20] Ekholm O, Kjøller M, Davidsen M, Hesse U, Eriksen L, Christensen A (2006). Danish Health and Morbidity Survey 2005 and trends since 1987.

[B21] Kjøller M, Rasmussen N (2002). Danish Health and Morbidity Survey 2000 and trends since 1987.

[B22] Videbæk J, Madsen M (2004). Heart Statistics.

[B23] Statistics Denmark (2000). Consumer study: private dental expenditures. Copenhagen.

[B24] Ministry of Interior and Health (2000). Dental Expenditures. Copenhagen.

[B25] The Danish Society of Nephrology (2000). National Registry – Report on Dialysis and Transplantation in Denmark 1999. Odense, Denmark.

[B26] Organisation of General Practitioners (2004). Cost study. Copenhagen.

[B27] Kristensen JK, Sandbaek A, Bro F, Lassen JF, Lauritzen T (2004). Routine screening for diabetic eye complications in a population based cohort of 4.438 persons with type 2 diabetes in a Danish county. Dan Med Bull.

[B28] Gislason GH, Abildstrom SZ, Rasmussen JN, Rasmussen S, Buch P, Gustafsson I, Friberg J, Gadsboll N, Kober L, Stender S, Madsen M, Torp-Pedersen C (2005). Nationwide trends in the prescription of beta-blockers and angiotensin-converting enzyme inhibitors after myocardial infarction in Denmark, 1995–2002. Scand Cardiovasc J.

[B29] (2005). Liquid-based and smear techniques used for cervical cancer screening in Denmark: a medical technology assessment.

[B30] Organisation for Economic Co-operation and Development (2004). The OECD Health Project: Towards High-Performing Health Systems Paris.

[B31] Organisation for Economic Development and Co-operation (2005). OECD Health Data 2005: Statistics and Indicators for 30 Countries. Paris.

[B32] The World Health Report (2000). Health systems: improving performance.

[B33] National Health Service Institute for Innovation and Improvement (2006). Improving care for people with long-term conditions: a review of UK and international frameworks. Warwick.

[B34] Fisher ES, Wennberg JE, Stukel TA, Skinner JS, Sharp SM, Freeman JL, Gittelsohn AM (2000). Associations among hospital capacity, utilization, and mortality of US Medicare beneficiaries, controlling for sociodemographic factors. Health Serv Res.

[B35] Popovic J, Hall M (2001). 1999 National Hospital Discharge Survey. Advance Data.

[B36] Gatsonis CA, Epstein AM, Newhouse JP, Normand SL, McNeil BJ (1995). Variations in the utilization of coronary angiography for elderly patients with an acute myocardial infarction. An analysis using hierarchical logistic regression. Med Care.

[B37] Wennberg JE, Peters PG (2004). Unwarranted variations in the quality of health care: can the law help medicine provide a remedy/remedies?. Spec Law Dig Health Care Law.

[B38] Kramers PG (2003). The ECHI project: health indicators for the European Community. Eur J Public Health.

[B39] Sant M, Aareleid T, Berrino F, Bielska Lasota M, Carli PM, Faivre J, Grosclaude P, Hedelin G, Matsuda T, Moller H, Moller T, Verdecchia A, Capocaccia R, Gatta G, Micheli A, Santaquilani M, Roazzi P, Lisi D (2003). EUROCARE-3: survival of cancer patients diagnosed 1990–94–results and commentary. Ann Oncol.

[B40] Veillard J, Champagne F, Klazinga N, Kazandjian V, Arah OA, Guisset AL (2005). A performance assessment framework for hospitals: the WHO regional office for Europe PATH project. Int J Qual Health Care.

[B41] Mattke S, Epstein AM, Leatherman S (2006). The OECD Health Care Quality Indicators Project: history and background. Int J Qual Health Care.

[B42] Mangione CM, Gerzoff RB, Williamson DF, Steers WN, Kerr EA, Brown AF, Waitzfelder BE, Marrero DG, Dudley RA, Kim C, Herman W, Thompson TJ, Safford MM, Selby JV (2006). The association between quality of care and the intensity of diabetes disease management programs. Ann Intern Med.

[B43] Diderichsen F, Preker A (2004). Resource allocation for health equity: issues and methods. Health, Nutrition, and Population Discussion Papers.

[B44] Deber R, Swan B (1999). Canadian health expenditures: where do we really stand internationally?. CMAJ.

[B45] Klauvus J, Linna M (2004). International comparisons of health expenditure: a serious policy-tool?. Global Forum for Health Research Mexico City.

[B46] Gosden TB, Torgerson DJ (2002). Economics notes: Converting international cost effectiveness data to UK prices. BMJ.

[B47] Organisation for Economic Co-operation and Development (2004). OECD Health Data 2004. Paris.

[B48] Talbot-Smith A, Gnani S, Pollock AM, Gray DP (2004). Questioning the claims from Kaiser. Br J Gen Pract.

[B49] Anderson GF, Reinhardt UE, Hussey PS, Petrosyan V (2003). It's the prices, stupid: why the United States is so different from other countries. Health Affairs.

[B50] Organisation for Economic Co-operation and Development (2008). OECD Health Data 2008: Statistics and Indicators for 30 Countries. Paris.

[B51] Fireman B, Bartlett J, Selby J (2004). Can disease management reduce health care costs by improving quality?. Health Affairs.

