# Supplementary material for: A retrospective analysis of health systems in Denmark and Kaiser Permanente
Source: BMC Health Serv Res. 2008 Dec 11;8:252. doi: 10.1186/1472-6963-8-252 (PMC2630928; doi:10.1186/1472-6963-8-252)
Supplement: Additional file 1 — Elements of health systems useful for making meaningful comparisons. Recommended sample types of information in six domain areas for comparing health systems. [file 1472-6963-8-252-S1.pdf]

Table 5 Elements of health systems useful for making meaningful comparisons

Dimension	Recommended sample types of information for comparisons	Dimension	Recommended sample types of information for comparisons
Population	• Individual level socio-demographic characteristics, eg, age, race/ethnicity<br>• Individual level socio-economic characteristics, eg, educational attainment, income, wealth<br>• Individual level health behaviour characteristics, eg, smoking, chemical dependency, obesity, diet<br>• Life expectancy<br>• Patterns of relevant chronic and acute conditions<br>• Relevant cultural factors, eg, gender equality, expectations and norms for health care use	Utilisation Patterns	• Average length of stay<br>• Specialty procedure rates<br>• Number of hospitals, total number of hospital beds, hospital bed occupancy rates<br>• Additional types of facilities, e.g., skilled nursing, rehab<br>• Technology availability and use, e.g., PET/MRI/CT scanners<br>• Primary care and specialty care visit rates
Professional Staff	• Physician supply: total, specialty, and generalist/primary care<br>• Nurse supply<br>• Supply of other health professionals, eg, physiotherapists, health behaviourists<br>• Roles and scope of practice<br>• Educational preparation for health professionals<br>• Competencies<br>• Specialisation demands	Quality	• Process measures, eg, screening rates<br>• Patient satisfaction<br>• Process, structure, and outcomes measures<br>• Use of evidence-based practices<br>• Care wait times<br>• Adverse event rates<br>• Perceived quality
Delivery System	• Organisational structure and governance<br>• Reimbursement/payment systems<br>• Local variations in organisational patterns<br>• Service delivery model<br>• Economic and non-economic incentives<br>• Continuity of care between settings and over time<br>• Supports for evidence-based practices<br>• Information technology	Costs	• PPP per capita health care costs<br>• Gross expenditures<br>• Payment schemes<br>• Benefit and entitlements<br>• Alternative adjustment methodologies<br>• Sources of funding<br>• Operating expenditures<br>• Unit cost and units consumed
